# T cells responding to *Trypanosoma cruzi* detected by membrane TNF‐α and CD154 in chagasic patients

**DOI:** 10.1002/iid3.197

**Published:** 2017-10-01

**Authors:** Juan G. Ripoll, Nicolás A. Giraldo, Natalia I. Bolaños, Nubia Roa, Fernando Rosas, Adriana Cuéllar, Concepción J. Puerta, John M. González

**Affiliations:** ^1^ Grupo de Ciencias Básicas Médicas, Facultad de Medicina Universidad de los Andes Bogotá Colombia; ^2^ Facultad de Medicina Pontificia Universidad Javeriana and Hospital Universitario San Ignacio Bogotá Colombia; ^3^ Clínica Abood‐Shaio Bogotá Colombia; ^4^ Grupo de Inmunobiología y Biología Celular, Facultad de Ciencias Pontificia Universidad Javeriana Bogotá Colombia; ^5^ Laboratorio de Parasitología Molecular, Facultad de Ciencias Pontificia Universidad Javeriana Bogotá Colombia

**Keywords:** CD4+ T lymphocytes, CD8+ T lymphocytes, chagas disease, flow cytometry, T lymphocyte

## Abstract

**Introduction:**

Chagas disease is a parasitic infection whose pathogenesis is related to parasite persistence and a dysfunctional cellular immune response. Variability in cytokine secretion among chronic *Trypanosoma cruzi*‐infected patients might preclude the identification of the pool of antigen specific T cells. The goal of this study was to determine the fraction of T cells responding to *T. cruzi* antigen measured by the expression of membrane TNF‐α and CD154.

**Methods:**

A total of 21 chagasic patients, 11 healthy and 5 non‐chagasic cardiomyopathy controls were analyzed. PBMCs were short‐term cultured in the presence of anti‐CD28, anti‐CD49d, anti‐TNF‐α, and TACE (TNF‐α converting enzyme) inhibitor either under *T. cruzi‐*lysate or polyclonal stimuli. Cells were stained with anti‐CD3, anti‐CD4, anti‐CD8, and anti‐CD154, and analyzed with flow cytometry.

**Results:**

CD4+ and CD8+ T cells in chagasic patients displayed higher percentages of membrane‐bound TNF‐α+ and CD154+ compared with controls after *T. cruzi*‐antigen stimulation. Both markers displayed a positive correlation in the T cell subpopulations analyzed. Symptomatic chagasic patients were differentiated from asymptomatic patients based on the expression of CD154 and membrane TNF‐α in TCD4+ and TCD8+ compartments, respectively.

**Conclusions:**

These results show that both markers could be useful for assessing the pool of antigen‐specific T cells in chronic chagasic patients.

## Introduction

TCR engagement is followed by the activation of an intracellular signaling cascade, which triggers T‐cell effector functions, including cytokine secretion and release of intracellular cytotoxic granules 1. Antigen elimination after acute infection is accompanied by apoptosis of the vast majority of effector T cells. Nonetheless, a fraction of antigen‐specific lymphocytes acquires a long‐lived memory phenotype that allows for a rapid and efficient immune cell response upon reinfection 2. Although memory immune response is committed to protection, persistent infections can result in T cell over‐activation and subsequent inefficient or pathogenic immune responses 3. Therefore, the assessment of cellular phenotype, effector function and frequency of antigen‐specific T cells is essential to better understand their protective or pathogenic role in chronic infectious diseases 4.

IFN‐γ secretion by CD8+ T cells has been commonly used as a read‐out to quantify antigen‐specific immune responses in both viral 5, 6 and parasitic diseases 7, 8. However, in chronic parasitic infections, IFN‐γ expression significantly fluctuates 7, 8, 9, 10. Indeed, the impairment of IFN‐γ secretion is part of the “T cell exhaustion” phenomena in which cellular functions such as cytotoxic capacity, cytokine production, and proliferative potential are sequentially diminished as a result of continuous antigen exposure 11, 12. Therefore, in chronic viral diseases, cytokines other than IFN‐γ have been commonly used to assess T‐cell antigen specificity according to the infection phase 13.

Chagas disease is a parasitic infection caused by a hemoflagellate protozoan named *Trypanosoma cruzi*. The acute phase of the infection is followed by a chronic phase with the presence of a low level parasitemia. CD4+ and CD8+ T lymphocytes participate in the control but not the elimination of *T. cruzi*, as consistently demonstrated in animal models and human infection 14, 15, 16. Approximately 30% of chronically infected individuals become symptomatic due to heart or gastrointestinal involvement 17. Nevertheless, the mechanisms of tissue damage in chronic Chagas disease still remain unclear. To date, parasite genotypes, antigen persistence, and dysfunctional cellular immune responses are among the proposed mechanisms 18. Indeed, heart biopsies from chronic chagasic patients show endomyocardial cell infiltration with a predominance of T cells 19, while parasites are rarely found in autopsy tissue samples 20.

In chronic Chagas disease, *T. cruzi*‐specific T cell function is altered 21, 22, 23. T cell IFN‐γ secretion is diminished as cardiac disease progresses, rendering it necessary to identify other markers of antigen T cell specificity 7, 24. Membrane TNF‐α has been used to assess antigen specific CD8+ T cells in viral models 25. Conversely, CD154 (CD40L) has been broadly used to identify antigen*‐*specific CD4+ T cells during bacterial and fungal infections 26, 27. Detection of both surface markers might be a useful alternative for antigen‐specific T cell recognition in parasitic models. Therefore, the aim of this study is to assess the use of membrane TNF‐α and CD154 as markers of T cells responding to *T. cruzi* antigen in chronic chagasic patients. TNF‐α has been widely implicated in the pathogenesis of Chagas disease 28 and used as an indicator of T cell function when detected intracellularly or in sera. However, neither membrane TNF‐α nor CD154 have been explored as markers of antigen‐specific T cell recognition during chronic *T. cruzi* infection 10, 29.

## Materials and Methods

### Ethics statement

The informed consent and the research protocol were approved by Ethical Committees from: Pontificia Universidad Javeriana (01–2010), Universidad de los Andes (039–2009), Hospital Universitario San Ignacio (77–2011), and Fundación Abood Shaio (134–2010), all in Bogotá, Colombia. All participants signed the written informed consent. This research followed Colombia national regulations and the Declaration of Helsinki.

### Selection of study population

Thirty‐seven volunteers were enrolled in the study and classified into three groups: healthy controls (HC), non‐chagasic cardiomyopathy (NCC), and chagasic patients (CP). For HC and NCC, the inclusion criteria were as follows: (1) not having lived or visited Chagas‐endemic areas for more than 6 months; (2) not having an immune‐related disease; (3) free of viral or infectious disease at least 2 weeks before the blood sampling; (4) two negative serological tests for anti‐*T. cruzi* antibodies, and for NCC donors; (5) confirmed diagnosis of non‐infectious cardiomyopathy. Healthy donors included two males and nine females ranging from 24 to 67 years old (43.82 ± SD 14.29), and the NCC donors included five patients, two males, and three females ranging from 51 to 63 years old (58.4 ± SD 4.67). Patients with Chagas disease were reactive by ELISA test and immune‐fluorescence assay test (IFAT); assays were performed at the National Institute of Health (INS) in Bogotá, Colombia. Patients were clinically evaluated and classified according to the American College of Cardiology/American Heart Association staging 30 as follows: five individuals in group A, four in group B, seven in group C, and five in group D. Patients were further subdivided into asymptomatic (Asympt, corresponding to A and B groups) and symptomatic chagasic cardiomyopathy groups (Sympt, groups C and D), Table 1.

**Table 1:** Baseline demographics and clinical characteristics of the individuals studied

	Asympt chagasic patients	Sympt chagasic patients	Healthy controls	Non‐chagasic cardiomyopathy
Number of individuals	9	12	11	5
Age media (±SD)	48.78 (±8.48)	54.08 (±9.37)	43.82 (±14.29)	58.40 (±4.67)
Female sex (%)	66.67	50	81.82	40
Clinical assessment				
ACC/AHAS classification				
A No. (%)	5 (55.56)	—	—	—
B No. (%)	4 (44.44)	—	—	—
C No. (%)	—	7 (58.33)	—	2 (40)
D No. (%)	—	5 (41.67)	—	3 (60)
Mean LVEF (%) (±SD)*	60 (±5.90)	38.33 (±17.49)	—	39 (±16.73)
Heart failure etiology				
Ischemic heart failure	—	—	—	2
Hypertensive heart failure	—	—	—	1
Dilated idiopathic heart failure	—	—	—	1
Valvular heart failure	—	—	—	1

Groups: Asympt chagasic patients: those with non‐structural cardiac damage (groups A and B). Sympt chagasic patients: those with structural cardiac damage (groups C and D). ACC/AHAS Classification: A: Normal electrocardiogram (ECG) and echocardiogram findings, and New York heart association functional classification (NYHA) I; B: abnormal ECG findings, normal ECHO and NYHA I; C: Abnormal ECG findings, increased heart size, decreased LVEF, and NYHA II or III; and D: same as class C, but NYHA IV. LVEF: left ventricular ejection fraction. *p‐value: 0.0683.

### Separation of peripheral blood mononuclear cells (PBMC)

Blood samples were obtained by venipuncture into heparinized tubes (BD Bioscience, Franklin Lakes, NJ). PBMC were isolated by Ficoll‐Hypaque density gradient centrifugation (Sigma–Aldrich, St. Louis, MO) and then gently re‐suspended in RPMI 1640 medium (Sigma–Aldrich). Some experiments were performed with frozen (PBMC) cells (RPMI medium with 10% of FCS and 10% of DMSO). Briefly, cells were thawed in the presence of nuclease inhibitor Benzonase 250 U/μl (Novagen, San Diego, CA). Trypan blue exclusion was used for the assessment of cell viability; the viability was higher than 85% in the frozen cells.

### CD154 and membrane TNF‐α labeling and analysis

A total of 3 × 10^6^ cells were incubated for 4.5 h at 37°C with 5% CO_2_ and equally distributed in three 15 ml conical tubes with 1 μg/ml anti‐CD28, 1 μg/ml anti‐CD49d, 0.5 mg/ml anti‐TNF‐α Pe‐Cy7 (BD Pharmingen, San Diego, CA) and 2.5 μl of 10 μM TNF‐α converting enzyme (TACE or ADAM 17) inhibitor or TAPI‐0 (Calbiochem; Cat. No. 579050; San Diego, CA, USA) under the following conditions: (1) cells in RPMI 1640 media alone; (2) 10 μg/ml of *T. cruzi* trypomastigote‐lysate 23; and (3) 3.7 µg/ml of Staphylococcal enterotoxin B (SEB). After culture, cells were washed with 0.01 M PBS pH 7.4 (or PBS 1×) followed by surface staining with anti‐CD3 APC (clone SK7), anti‐CD4 PerCP (SK3), CD8‐APCH7 (SK1), anti‐CD154 PE (TRAP1). All monoclonal antibodies were purchased from BD Bioscience (San Jose, CA). Samples were incubated at 4°C for 20 min in darkness with an additional 5 min of staining with 5 μl of propidium iodide (PI solution 50 μg/ml). Samples were acquired in a FACsCanto II with FACsDiva Software (BD Bioscience) and the data were analyzed with FlowJo version 4.2 (Tree Star Inc. Ashland, OR). At least 5 × 10^4^ cells were acquired in the lymphocyte population according to forward scatter (FSC) versus side scatter (SSC) features. Analyses were performed on live/CD3+ cells. Subpopulations of T cells were separated as follows: CD4+/TNFα+ and CD8+/TNFα+. A similar analysis was performed for CD154 surface expression, Figure S1. Flow cytometry data files are available for analysis in Flow Repository (https://flowrepository.org/), Repository ID: FR‐FCM‐ZY36.

### Co‐expression of CD107a/b and membrane TNF‐α in CD8+ T lymphocytes after *T. cruzi*‐derived antigen stimulation

Degranulation as readout for antigen‐specific CD8+ T cell activation was carried out by flow cytometry measuring surface expression of granule‐derived proteins CD107a and CD107b 31. Anti‐CD107a and CD107b FITC (clones H4A3 and H4B4, respectively) were added (10 µl each) immediately after stimulation according to the protocol described for membrane TNF‐α staining. Monoclonal antibodies were purchased from BD Bioscience. Gating for CD107a and CD107b expression was performed based on isotype controls.

### TCR‐Vβ flow cytometry staining

TCR‐Vβ repertoire was analyzed by flow cytometry, using the IOTest Beta Mark Kit (Beckman‐Coulter) to stain 1 × 10^6^ cells/tube, and run following manufacturer's instructions. T cells staining panel was similar to the one described for CD154 and membrane TNF‐α. Antibody for TCR‐Vβ was PE labeled and TCR expression was determined in the gate of TNF‐α positive cells (Fig. S2).

### Statistical analysis

Statistical analysis was performed using GraphPad Prism version 6.0 (GraphPad Software, San Diego, CA). Comparisons between two groups were assessed using the Mann–Whitney U‐test (2‐tailed). A one‐way ANOVA non‐parametric Kruskal–Wallis test (2‐tailed) with Dunn's test was used for multiple comparisons. Spearman's correlation test and linear regression were used to measure the correlation between CD154 and membrane TNF‐α variables in T CD4+ and T CD8+ lymphocytes. *p* values less than <0.05 were considered statistically significant.

## Results

### Membrane TNF‐α and CD154 expression in non‐stimulated and polyclonal stimulated T cell subpopulations

**Figure 1:**
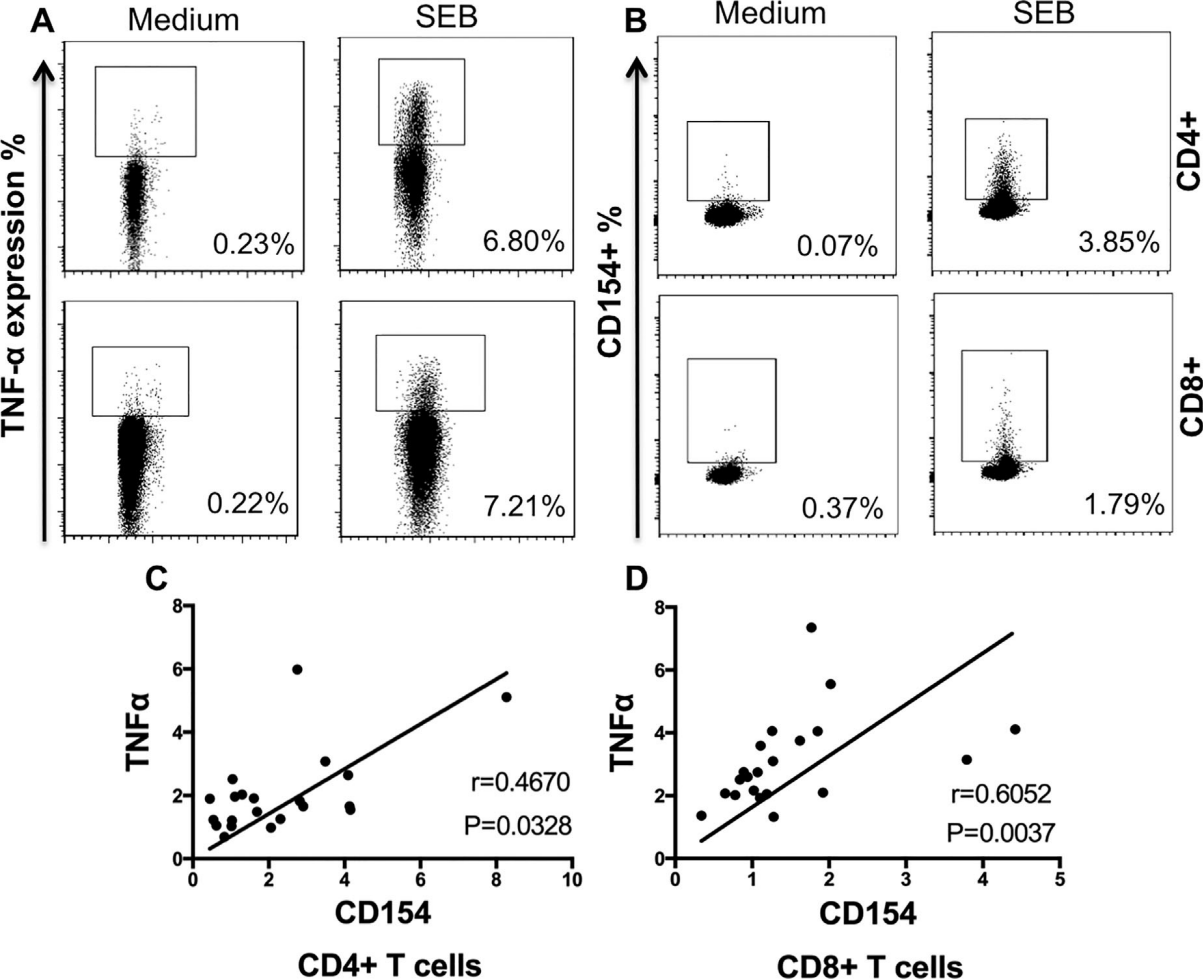
CD154 and membrane TNF‐α expression among T cell subsets. PBMC from chronic chagasic patients were cultured for 4.5 h and stimulated under the following conditions: polyclonal stimuli or medium alone. Relative size or FSC is displayed in *x*‐axis. Examples of dots plot for membrane TNF‐α (A) and CD154 expression (B) in CD4+ (upper panel) and CD8+ (lower panel) T cells under medium alone and SEB as indicate in *y*‐axis. Scatter plot showing correlation of membrane TNF‐α and CD154 on CD4+ T cells (C) and CD8+ T cells (D) in cells from chagasic patients.

The expression of both surface markers in the T cell subsets increased after polyclonal stimulus compared with non‐stimulated cells (Figs. 1A and 1B; Table 2). No differences were identified when fresh ex vivo PBMC or frozen cells were used (Table S1). T cell expansion under polyclonal stimulus indicated that cells from all donors were competent in secretion of TNF‐α after short‐term stimulation (Table 2).

**Table 2:** Expression of membrane TNF‐α and CD154 on T cells cultured in media or polyclonal stimuli

	Asymptomatic chagasic patients		Symptomatic chagasic patients		Healthy controls (HC)		Non‐chagasic cardiomyopathy (NCC)	
	Medium	SEB	Medium	SEB	Medium	SEB	Medium	SEB
Membrane TNF‐α								
CD4+ T cells (% Median & IQR)	0.60 (0.53–0.72)	5.17 (4.94–6.80)	0.40 (0.38–0.75)	4.65 (3.45–6.69)	0.30 (0.29–0.40)	5.07 (3.87–6.56)	0.57 (0.38–0.60)	8.51 (7.06–9.10)
CD8+ T cells (% Median & IQR)	0.79 (0.37–1.03)	6.47 (3.97–11.67)	0.50 (0.29–0.72)	6.94 (4.96–11.12)	0.35 (0.32–0.42)	5.44 (3.51–10.90)	0.97 (0.41–1.04)	13.13 (4.64–15.06)
CD154 (CD40L)								
CD4+ T cells (% Median & IQR)	0.28* (0.11–0.43)	9.55 (5.23–14.09)	0.32* (0.18–1.17)	5.35 (4.44–7.37)	0.10 (0.03–0.13)	4.23 (3.22–6.75)	0.09 (0.08–0.10)	11.83 (10.04–13.80)
CD8+ T cells (% Median & IQR)	0.19 (0.15–0.30)	1.35 (0.97–1.63)	0.23 (0.13–0.68)	1.82 (1.25–2.49)	0.14 (0.06–0.21)	0.84 (0.42–1.30)	0.24 (0.11–0.31)	0.80 (0.53–1.03)

SEB, Staphylococcal enterotoxin B.

Data are shown as median and interquartile range (IQR).

* *p* = 0.045 compared to healthy controls and non‐chagasic cardiomyopathy.

Although the differences were not statistically significant, T cells from non‐chagasic cardiomyopathy donors (NCC) tended to have higher expression of membrane TNF‐α after SEB stimulus than the other groups. Upon polyclonal stimulus, the expression of CD154 was higher in CD4+ than in CD8+ T cells from all groups. CD4+/CD154+ T cells without stimulation were higher in chagasic patients compared to healthy controls (HC) and NCC, Kruskal–Wallis, *p* = 0.0045, Table 2. A subsequent analysis was carried out in cells after parasite‐derived antigenic stimulation. To compare our results with data from previous studies, values from all chagasic patients are presented below as mean percentages and standard deviation: expression of membrane TNF‐α was 2.04% ± 1.31 for CD4+ and 3.06% ± 1.43 for CD8+ T cells; whereas for CD154+ mean membrane TNF‐α expression was 2.30% ± 1.84 for CD4+ and 1.48% ± 0.98 for CD8+ T cells. CD154 and membrane TNF‐α in both CD4+ T cells and CD8+ T cells positively correlated in all chronic chagasic patients tested, Figure 1C (Spearman *r* = 0.4670, *p* = 0.0328) and Figure 1D (Spearman, *r* = 0.6052, *p* = 0.0037), respectively. The following sections describe the features of each subpopulation of T cells independently.

### Membrane TNF‐α on CD4+ T cells stimulated with *T. cruzi* antigen

**Figure 2:**
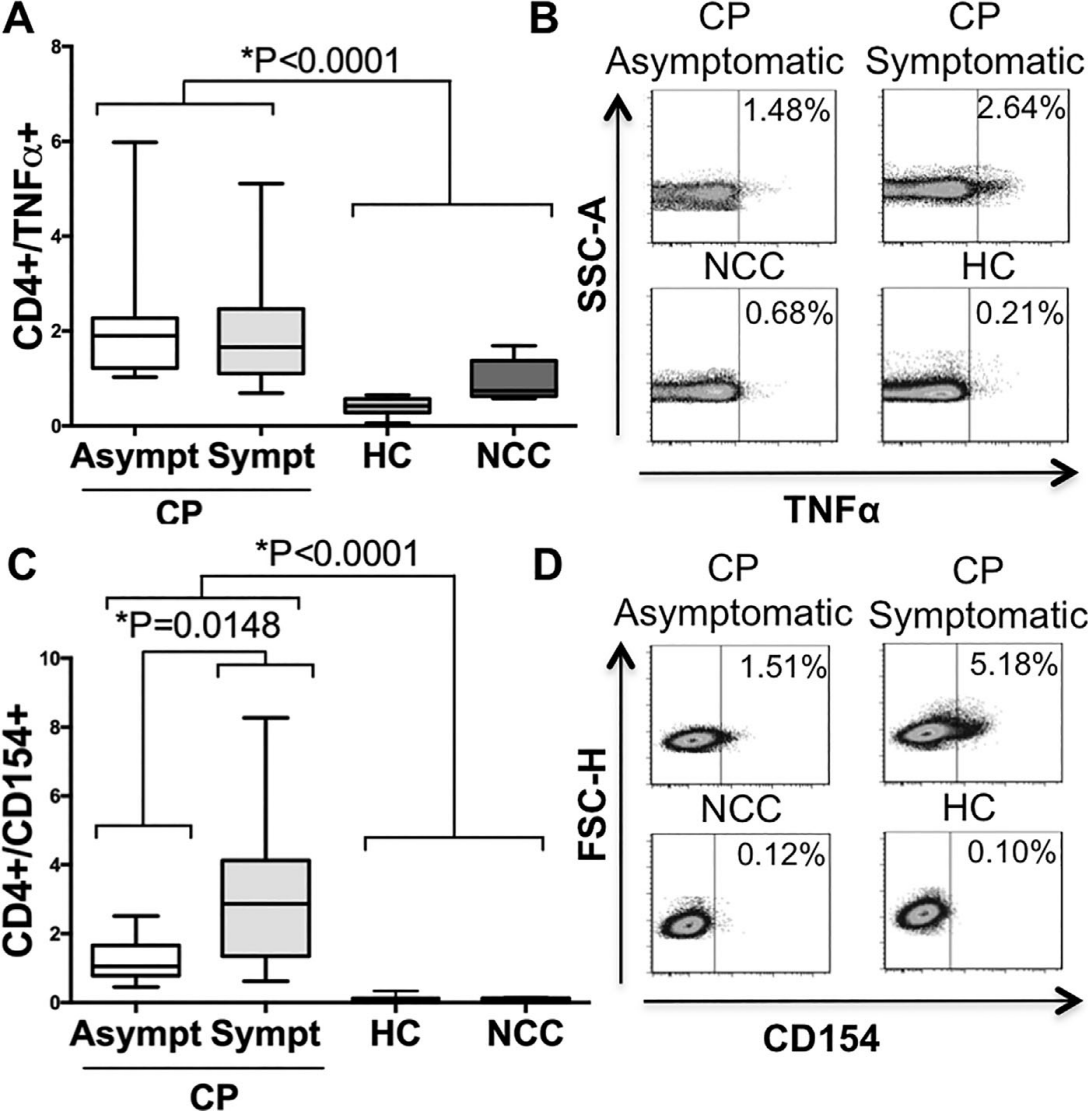
Membrane TNF‐α expression and CD154 on CD4+ T cells after *Trypanosoma cruzi* antigen‐derived stimulus. Percentage of membrane TNF‐α expression for CD4+ T cells in the analyzed groups (A). Examples of density/scatter plots for membrane TNF‐α (*x*‐axis) and CD4+ T cells (B). Percentage of CD154 expression for CD4+ T cells on the analyzed groups (C). Examples of density/scatter for CD154 (*x*‐axis) and CD4+ T cells (D). Box and whiskers indicate the median frequency and range of CD154 and membrane TNF‐α expression among CD4+ T cells (25th–75th percentile). CP, Chagasic patient; CP Asympt, Asymptomatic Chagasic Patients; CP Sympt, Symptomatic Chagasic Patients; HC, Healthy Controls; and NCC, Non‐Chagasic Cardiomyopathy.

Membrane‐bound TNF‐α has been used as a marker for detection of antigen‐specific CD8+ T cells in human viral infections 25. In CD4+ T cells from symptomatic (median 1.66%—IQR 1.21–2.13) and asymptomatic CP (1.90—IQR 1.23–2.03) patients, the percentage of TNF‐α+ cells increased after *T. cruzi* lysate stimulus when compared with NCC (0.74—IQR 0.68–1.06) or HC (0.42—IQR 0.32–0.53), Kruskal–Wallis, *p *< 0.0001, Figure 2A. Examples of flow cytometry dot plots are shown in Figure 2B. There was no difference in CD4+/TNF‐α+ T cells in the presence of parasite lysate among symptomatic and asymptomatic chagasic patients, Mann–Whitney *U* test, *p* = 0.7939.

### CD154 (CD40L) as marker of *T. cruzi* antigenic activation of CD4+ T cells

CD154 has been considered a marker of antigen‐specific CD4+ T cells in fungal, bacterial and viral infections 26, 27. After whole‐parasite antigen stimulation, the percentage of CD4+/CD154+ T lymphocytes was higher in symptomatic CP (median 2.87%, IQR = 1.82–4.10) and asymptomatic CP (1.05—IQR 1.02–1.61) compared to NCC (0.09—IQR 0.07–0.12) and HC donors (0.07—IQR 0.06–0.13), Kruskal–Wallis, *p *< 0.0001, Figure 2C. There was a difference in the percentage of CD4+/CD154+ T cells in chagasic donors according to the presence or absence of symptoms, Mann–Whitney *U* test *p* = 0.0148, Figure 2C. Examples of flow cytometry dot plots are shown in Figure 2D.

### Membrane TNF‐α expression on CD8+ T cells stimulated with *T. cruzi* antigen

**Figure 3:**
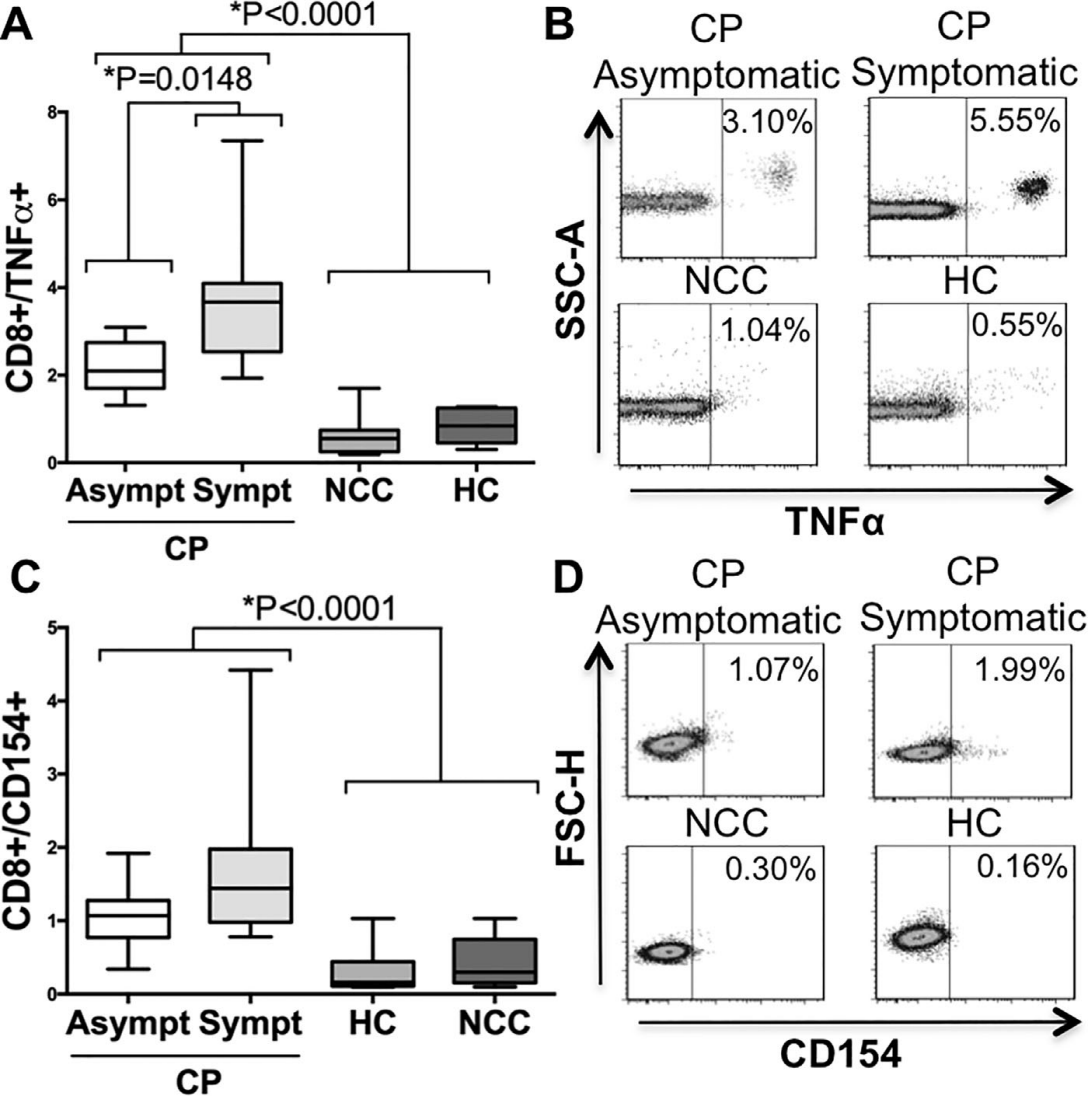
Membrane TNF‐α and CD154 expression on CD8+ T cells after *T. cruzi* antigen‐derived stimulus. Percentage of membrane TNF‐α+ expression for CD8+ lymphocytes in the analyzed groups (A). Examples of density/scatter plots for membrane TNF‐α (*x*‐axis) and CD8+ T cells (B). Percentage of CD154 expression for CD8+ T cells in the analyzed groups (C). Examples of density/scatter for CD154 (*x*‐axis) and CD8+ T cells (D). Box and whiskers indicate the median frequency and range of CD154 and membrane TNF‐α expression among CD8+ T cells (25th–75th percentile). CP, Chagasic patient; CP Asymptomatic, Asymptomatic Chagasic Patients; CP Symptomatic, Symptomatic Chagasic Patients; HC, Healthy Controls; and NCC, Non‐Chagasic Cardiomyopathy.

The percentage of T CD8+/TNF‐α+ cells after parasite‐derived antigen exposure, was higher in both symptomatic (median 3.67%—IQR 2.58–4.07) and asymptomatic (2.10—IQR 2.05–2.74) CP compared to NCC (0.84—IQR 0.6–1.22) and HC (0.55—IQR 0.28–0.72), Kruskal–Wallis, *p *< 0.0001, Figure 3A. Examples of flow cytometry dot plots are shown in Figure 3B. Moreover, symptomatic CP displayed higher frequencies when compared with asymptomatic CP, Mann–Whitney *U* test *p* = 0.0148.

### CD154 (CD40L) as marker of *T. cruzi* antigenic activation of CD8+ T cells

Symptomatic (median 1.07%—IQR 0.89–1.27) and asymptomatic CP (1.44—IQR 1.06–1.89) had higher percentages of CD8+/CD154+ T cells after *T. cruzi* lysate stimulus compared to NCC (0.30—IQR 0.21–0.46) and HC (0.16—IQR 0.12–0.36), Kruskal–Wallis, *p *< 0.0001. T cell expression of CD154 in NCC and HC was similar to cell culture in media alone, Table 2. The percentage of CD154+ expressing cells and examples of flow cytometry dot plots are shown in Figures 3C and 3D, respectively. There was no difference in CD8+/CD154+ T cells between symptomatic and asymptomatic chagasic patients, Mann–Whitney *U* test *p* = 0.1694. A summary of the results for CD154 and membrane TNF‐α in CD4+ and CD8+ T cells is shown in Table S2.

### CD107a/b and membrane TNF‐α co‐expression in CD8+ T cells

**Figure 4:**
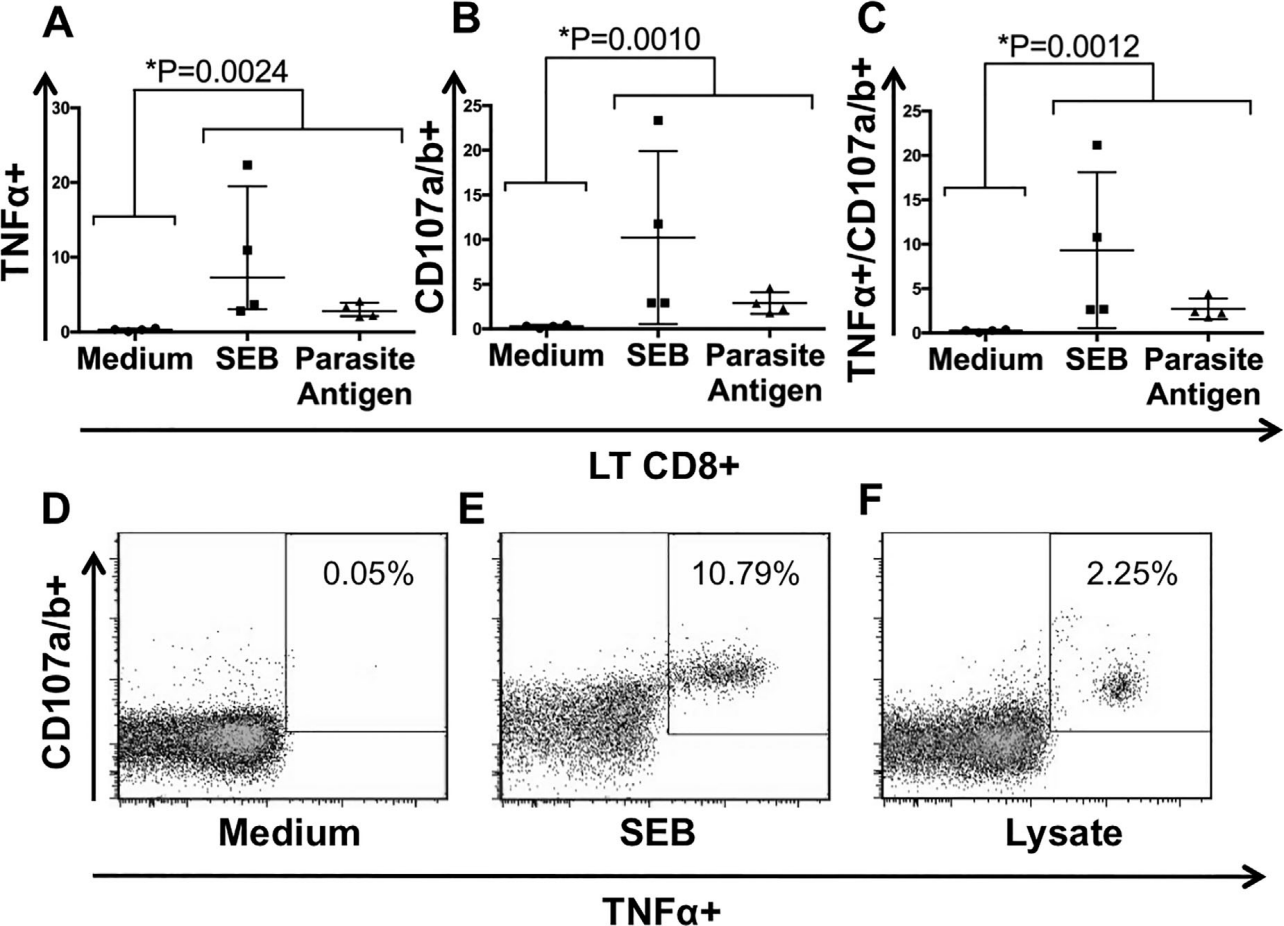
Expression and co‐expression of CD107a/b and membrane TNF‐α in CD8+ T lymphocytes. Percentage of membrane TNF‐α (A), CD107a/b (B) and both surface markers (C) in CD8+ T cells from chagasic patients under the following conditions: medium, *T. cruzi* lysate and SEB stimuli. Examples of cytometry plots of membrane TNF‐α and CD107a/b co‐expression under medium alone (D), SEB stimuli (E) and *T. cruzi* lysate exposure (F).

Surface detection of CD107a and CD107b is used to assess CD8+ T cell degranulation after antigenic activation 31. In this study, this surface marker was tested to confirm whether the detection of membrane TNF‐α is a useful marker of *T. cruzi*‐specific CD8+ T cells. Membrane TNF‐α and CD107a/b expression in CD8+ T cells was higher after polyclonal and whole parasite antigen stimulation when compared with cells in culture media, Kruskal–Wallis, *p *< 0.05 for both analyses, as shown in Figures 4A and 4B, respectively. Percentages of co‐expression of CD107a/b and membrane TNF‐α were very similar to each independent marker in all conditions assessed: *T. cruzi* antigenic stimulation (Median 2.33%—IQR 2.14–2.91), polyclonal stimulation (6.73—IQR 2.66–13.39) and culture media (0.29—IQR 0.22–0.32), Figure 4C. Examples of the dot plot for CD107a/b are shown in Figures 4D–F.

Regarding TCR‐Vβfamilies, T cells producing TNF‐α after stimulation with *T. cruzi* lysate showed more spread TCR usage among CD4+ (Fig. S2A and B) than CD8+ T cells (Fig. S2C and D). There was not a common pattern between the two donors tested. Nevertheless, despite TCR polyclonal responses, higher percentages were identified for TCR Vβ1, 16 and 4 in CD4+ T cells and TCR Vβ5.1, 13.6 and 17 in CD8+ T cells (Fig. S2).

## Discussion

Cytokine secretion can be limited by the T cell exhaustion state 12, 32 during chronic viral infections 33, 34, malignant tumors 35, and parasitic diseases 36. Chronic chagasic patients displayed dysfunctional T cell responses characterized by increased frequency of terminally differentiated cells, monofunctional antigen‐specific T cell responses and progressive attenuation of cytokine production 7, 10, 21, 37. IFN‐γ secretion in T cells decreased with the severity of cardiac disease in both 24 CD4+ 7 and CD8+ 37 T cell subsets. Indeed, the percentage of INF‐γ secretion by *T. cruzi‐*specific T cells in PBMC from nine chronic chagasic patients exposed to amastigote lysate ranged from 0.002% to 2.56% for CD8+ T cells and 0.014% to 1.57% for CD4+ T cells. However, the clinical status of chronic chagasic patients included in the study was not described 7.

A recent investigation used three intracellular cytokines (IFN‐γ, TNF‐α, or IL‐2) as markers of CD8+ T cell functionality 23. Upon parasite stimuli, asymptomatic chagasic patients displayed a higher percentage of bifunctional (IFN‐γ+/TNF‐α+) T cells, while symptomatic chagasic patients displayed a higher percentage of monofunctional T cells (IFN‐γ+ or TNF‐α+) 23. Regardless of the phenotype, TNF‐α seemed to be the cytokine most consistently secreted among T cell subpopulations in chronic chagasic patients 10, 23. Thus, similar to T cell exhaustion in viral models, IL‐2 secretion is hampered while TNF­­‐α is maintained, even in advanced phases of Chagas disease (cardiac manifestations) in which antigenic‐specific T cells displayed an effector rather than memory T cell phenotype 10, 23.

CD154 (CD40L) and membrane TNF‐α emerged as an alternative method to assess the frequency of CD4+ and CD8+ antigen‐specific T cells in bacterial, fungi and human viral infections 25, 26, 27. PMBCs stimulated with *T. cruzi*‐lysate from children without evidence of cardiac involvement reported that the pool of antigenic‐specific T cells was enriched with CD4+/CD154+ and CD4+/TNF‐α+ T cells 29. In an adult population, we obtained similar frequencies for CD4+/CD154+ (mean 2.04% for all chagasic patients) and CD4+/membrane TNF‐α+ T cells (mean 3.06% in symptomatic patients). Interestingly, the same study demonstrated that the fraction of antigen‐specific T cells measured by surface CD4+/CD154+ and intracellular CD4+/TNF‐α+ T cells was almost undetectable in chronic adult chagasic patients with mild cardiac disease 29. Differences between previous studies could be explained by the antigen used (amastigote or trypomastigotes), time of incubation and stimulation, and method of cytokine detection (intracellular vs. membrane).

In our study, we showed an increased fraction of T cells expressing membrane TNF‐α and CD154 after antigenic stimulation among chronic chagasic patients. Interestingly, CD4+/CD154+ and CD8+/TNFα+ T cell expression allowed for differentiation between asymptomatic and symptomatic chagasic patients. Indeed, both T cell subpopulations were augmented in patients with severe myocardiopathy. TNF‐α can bind its receptor as a soluble or membrane‐bound molecule activating different signaling pathways that will, in turn, produce different cellular responses 38. During *T. cruzi* infection in rodent models, TNF‐α secretion is triggered by parasites in macrophages and T cells 39. In combination with IFN‐γ, TNF‐α contributed to the control of intracellular parasites 40. Conversely, locally produced TNF‐α in infected hearts 41 has been associated with cardiac damage 42, progressive inflammation 43, and cardiomyocyte death 44. Notably, patients with severe chagasic cardiomyopathy (dysfunctional ventricular activity and disease progression) had higher levels of TNF‐α in serum than patients with mild disease 45. CD154 or CD40L is a member of the TNF superfamily and binds to the CD40 molecule on antigen presenting cells 27, 46. The interaction between CD40L and CD40 triggers adaptive immune response effector functions. Interestingly, in a rodent model of acute *T. cruzi* infection, CD40L ligation on immune cells using transferred‐CD40+ fibroblasts was associated with a reduction in parasitemia 47. We hypothesize that increased expression of CD154 (CD40L) among the chronic Chagas disease population might be the result of an adaptive immune response to parasite persistence.

Detection of anti‐*T. cruzi* specific T cells can be difficult in peripheral blood due to the heterogeneity of T cells population and the size of *T. cruzi* proteome with a high potential of HLA restricted T cells epitopes 7, 48. In a recent study, following *ex vivo* expansion of PBMCs from chagasic patients, IFN‐γ and GM‐CSF were considered the best read out of antigen specificity among cytokines tested in CD4+ T cells clones. Although in this study TNF‐α was measured, the data is not shown 48. In the same investigation, TCR usage in two CD4+ T cell lines displayed predominance of TCR‐Vβ 5.2 and 5.1 families 48. Interestingly, we identified the same TCR‐Vβ families among TNF‐α producing CD4+ and CD8+ T cells in two chagasic patients (Fig. S2).

In this study, the early secretion of TNF‐α in T cell subsets from chronic chagasic patients allowed the detection T cells responding to *T. cruzi* antigen. Expression of membrane TNF‐α correlated with an established surface marker (CD154) in CD4+ T cells and markers of degranulation (CD107a/b) among CD8+ T cells. In summary, membrane TNF‐α and CD154 could be useful markers to determine the fraction of antigen‐specific T cells in PBMC from chagasic patients stimulated with parasite lysate.

## Acknowledgments

The authors want to thank to Zulma Cucunubá MD for helping us with patient recruitment at Instituto Nacional de Salud (INS), Bogotá (Colombia), Juan Camilo Vargas‐Zambrano MD, M.Sc. for reviewing the manuscript and Sandra Quijano PhD, School of Sciences, Pontificia Universidad Javeriana in Bogotá, Colombia for her support with TCR flow cytometry assays.

## Conflict of Interest

None declared.

## Supporting information

Additional supporting information may be found in the online version of this article at the publisher's web‐site.

**Figure S1**. Cell gating strategy by flow cytometry.

**Figure S2**. Percentages of TCR‐Vβ families used by T cell subsets producing TNF‐α in response to *T. cruzi* lysate in two patients with chronic chagasic cardiomyopathy.

**Table S1**. Comparison of membrane TNF‐α and CD154 expression among *ex vivo* and frozen cells.

**Table S2**. Membrane TNF‐α and CD154 expression in T cell subsets after *T. cruzi* lysate stimulation.
