# Supplementary material for: T cells responding to Trypanosoma cruzi detected by membrane TNF‐α and CD154 in chagasic patients
Source: Immun Inflamm Dis. 2017 Oct 1;6(1):47–57. doi: 10.1002/iid3.197 (PMC5818450; doi:10.1002/iid3.197)
Supplement: Supplementary file 1 — Figure S1. Cell gating strategy by flow cytometry. [file IID3-6-47-s001.pdf]

T cells responding to *T. cruzi* detected by membrane TNF-α and CD154 in chagasic patients

Juan G. Ripoll[1], Nicolás A. Giraldo[1#a], Natalia I. Bolaños[1], Nubia Roa[2], Fernando Rosas[3], Adriana Cuéllar[4], Concepción J. Puerta[5], John M. González[1*]

[1] Grupo de Ciencias Básicas Médicas, Facultad de Medicina, Universidad de los Andes, Bogotá, Colombia. Address: Cra 1 # 18A-12. Phone Number: (571) 3394949 Ext. 3900.

[2] Facultad de Medicina, Pontificia Universidad Javeriana and Hospital Universitario San Ignacio, Bogotá, Colombia. Address: Cra. 7 No. 40-62. Phone number: (571) 320 8320 Ext. 2745 – 2777.

[3] Clínica Abood-Shaio, Bogotá, Colombia. Address: Dg 115a # 70c-75. Phone number: (571) 593 8210.

[4] Grupo de Inmunobiología y Biología Celular, Facultad de Ciencias, Pontificia Universidad Javeriana, Bogotá, Colombia. Address: Carrera 7 No. 43-82 – Building Carlos Ortíz. Phone number: (571) 320 8320 Ext. 4060 – 4134.

[5] Laboratorio de Parasitología Molecular, Facultad de Ciencias, Pontificia Universidad Javeriana, Bogotá, Colombia. Address: Carrera 7 No. 43-82 - Building Carlos Ortíz. Phone number: (571) 320 8320 Ext. 4060 – 4134.

[#a] Current address: INSERM UMRS872, Cancer, Immune Control and Escape, Cordeliers Research Center, Paris, France.

*** Corresponding author:**

John Mario González MD, PhD. School of Medicine, Universidad de los Andes, Bogotá D.C. Cra 1 # 18A-12. Zip code: 111711. Phone Number: 57 (1) 3394949 ext. 3900.

E-mail: johgonza@uniandes.edu.co (JMG)

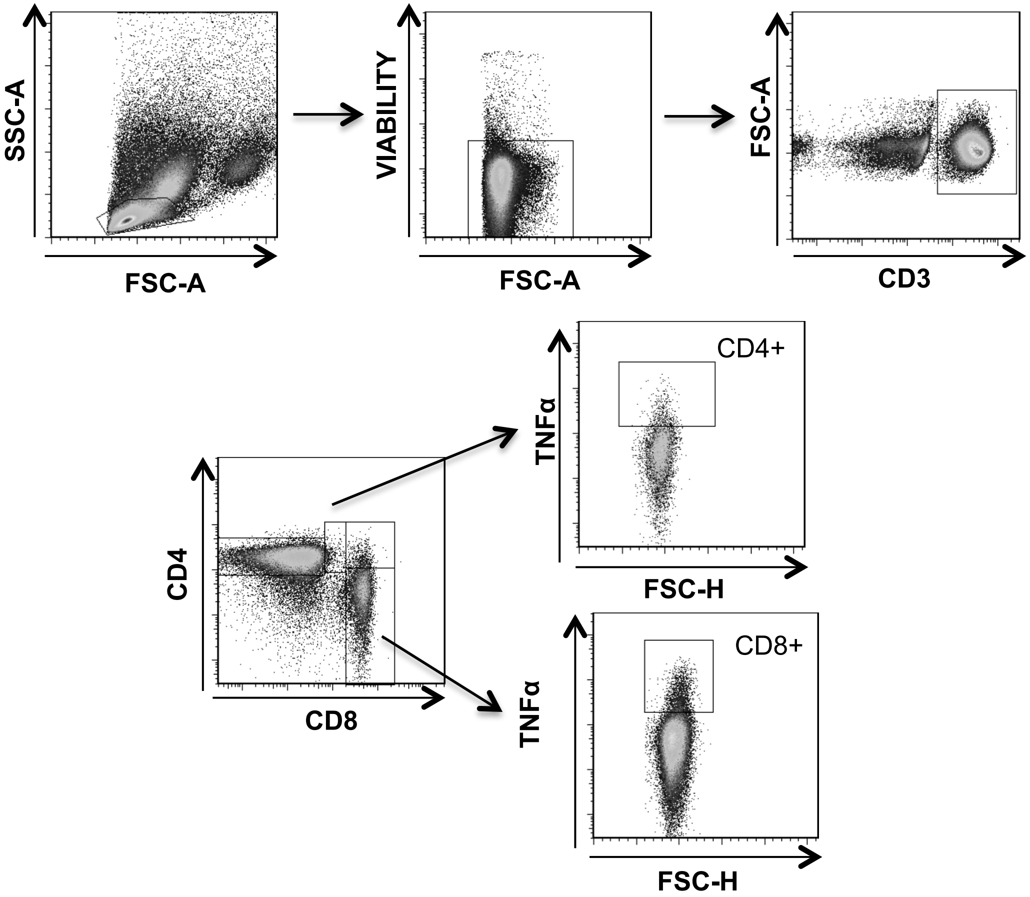
SSC-A
FSC-A
VIABILITY
FSC-A
FSC-A
CD3
CD4
CD8
TNFα
FSC-H
CD4+
TNFα
FSC-H
CD8+

Fig S1. Cell strategy gating by flow cytometry. Dot plots obtained from antigen-specific PBMC by flow cytometry. It shows the selection of the studied populations. Initially, cells were gated according FCS versus SSC characteristics (mononuclear cells), viability in PI negative cells and then selected by CD3+ expression (T cells). CD3+ cells were later divided into CD3+/CD4+ (T helper) and CD3+/CD8+ (cytotoxic T cells). Further analysis for CD154 and membrane TNF-α was performed in these T cell subsets.
