# Supplementary material for: T cells responding to Trypanosoma cruzi detected by membrane TNF‐α and CD154 in chagasic patients
Source: Immun Inflamm Dis. 2017 Oct 1;6(1):47–57. doi: 10.1002/iid3.197 (PMC5818450; doi:10.1002/iid3.197)
Supplement: Supplementary file 2 — Figure S2. Percentages of TCR‐Vβ families used by T cell subsets producing TNF‐α in response to T. cruzi lysate in two patients with chronic chagasic cardiomyopathy. [file IID3-6-47-s002.pdf]

T cells responding to *T. cruzi* detected by membrane TNF-α and CD154 in chagasic patients

Juan G. Ripoll[1], Nicolás A. Giraldo[1#a], Natalia I. Bolaños[1], Nubia Roa[2], Fernando Rosas[3], Adriana Cuéllar[4], Concepción J. Puerta[5], John M. González[1*]

[1] Grupo de Ciencias Básicas Médicas, Facultad de Medicina, Universidad de los Andes, Bogotá, Colombia. Address: Cra 1 # 18A-12. Phone Number: (571) 3394949 Ext. 3900.

[2] Facultad de Medicina, Pontificia Universidad Javeriana and Hospital Universitario San Ignacio, Bogotá, Colombia. Address: Cra. 7 No. 40-62. Phone number: (571) 320 8320 Ext. 2745 – 2777.

[3] Clínica Abood-Shaio, Bogotá, Colombia. Address: Dg 115a # 70c-75. Phone number: (571) 593 8210.

[4] Grupo de Inmunobiología y Biología Celular, Facultad de Ciencias, Pontificia Universidad Javeriana, Bogotá, Colombia. Address: Carrera 7 No. 43-82 – Building Carlos Ortíz. Phone number: (571) 320 8320 Ext. 4060 – 4134.

[5] Laboratorio de Parasitología Molecular, Facultad de Ciencias, Pontificia Universidad Javeriana, Bogotá, Colombia. Address: Carrera 7 No. 43-82 - Building Carlos Ortíz. Phone number: (571) 320 8320 Ext. 4060 – 4134.

[#a] Current address: INSERM UMRS872, Cancer, Immune Control and Escape, Cordeliers Research Center, Paris, France.

*** Corresponding author:**

John Mario González MD, PhD. School of Medicine, Universidad de los Andes, Bogotá D.C. Cra 1 # 18A-12. Zip code: 111711. Phone Number: 57 (1) 3394949 ext. 3900.

E-mail: johgonza@uniandes.edu.co (JMG)

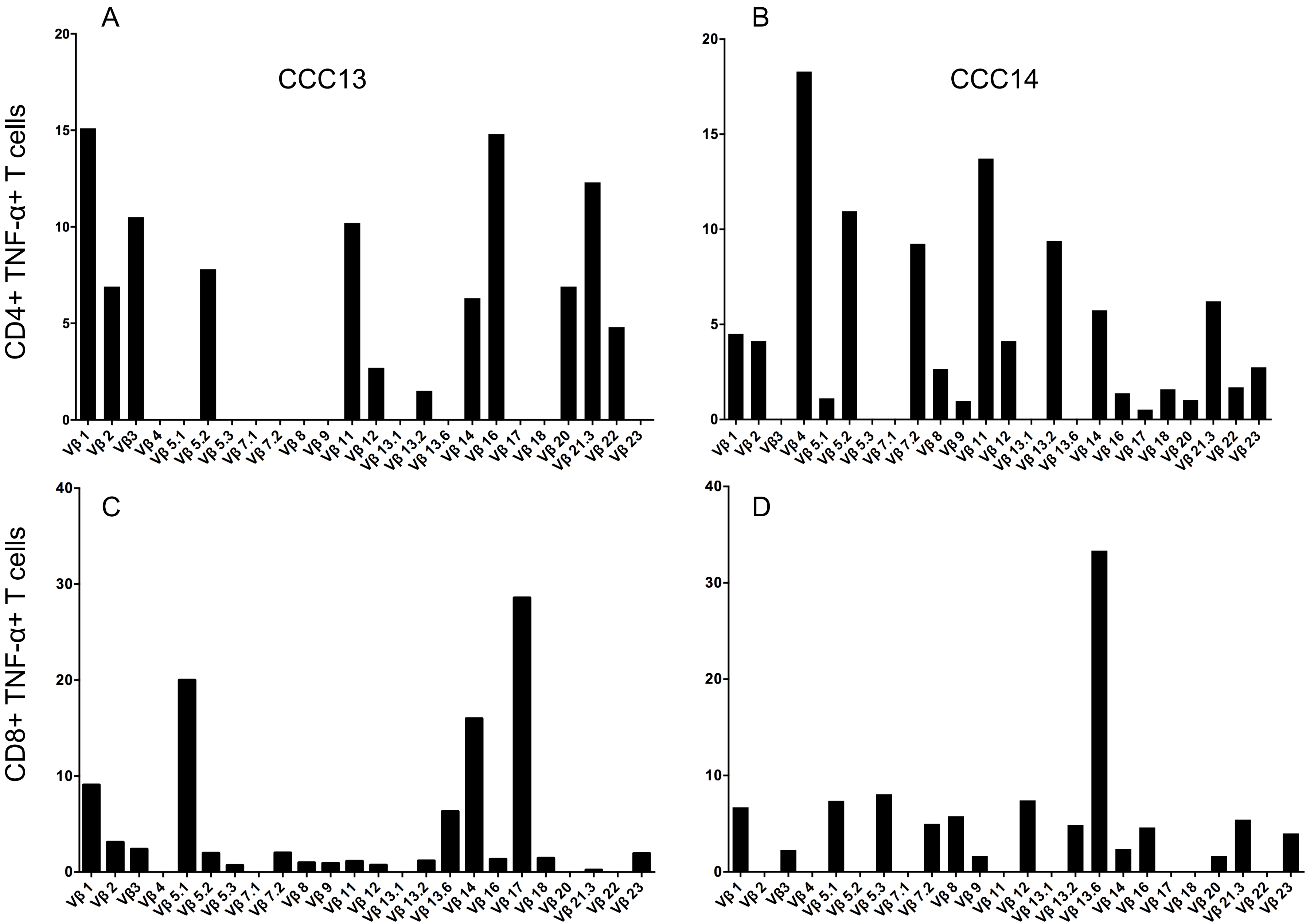

A
B
C
D
CCC13
CCC14
CD4+ TNF-α+ T cells
CD8+ TNF-α+ T cells
0
5
10
15
20
30
40
Vβ 1
Vβ 2
Vβ3
Vβ 4
Vβ 5.1
Vβ 5.2
Vβ 5.3
Vβ 7.1
Vβ 7.2
Vβ 8
Vβ 9
Vβ 11
Vβ 12
Vβ 13.1
Vβ 13.2
Vβ 13.6
Vβ 14
Vβ 16
Vβ 17
Vβ 18
Vβ 20
Vβ 21.3
Vβ 22
Vβ 23

Figure S2. Percentages of TCR-Vβ families used by T cell subsets producing TNF-α in response to *T. cruzi* lysate in two patients with chronic chagasic cardiomyopathy. Percentage of membrane TNF-α expression in CD4+ (A and B) and CD8+ T cells (C and D) for each TCR-Vβ family. Population is gated on CD3+CD4+/TNF-α+ and CD3+CD8+/TNF-α+, respectively. CCC= chronic chagasic cardiomyopathy.
