# Supplementary material for: T cells responding to Trypanosoma cruzi detected by membrane TNF‐α and CD154 in chagasic patients
Source: Immun Inflamm Dis. 2017 Oct 1;6(1):47–57. doi: 10.1002/iid3.197 (PMC5818450; doi:10.1002/iid3.197)
Supplement: Supplementary file 3 — Table S1. Comparison of membrane TNF‐α and CD154 expression among ex vivo and frozen cells. [file IID3-6-47-s003.pdf]

T cells responding to *T. cruzi* detected by membrane TNF-α and CD154 in chagasic patients

Juan G. Ripoll[1], Nicolás A. Giraldo[1#a], Natalia I. Bolaños[1], Nubia Roa[2], Fernando Rosas[3], Adriana Cuéllar[4], Concepción J. Puerta[5], John M. González[1*]

[1] Grupo de Ciencias Básicas Médicas, Facultad de Medicina, Universidad de los Andes, Bogotá, Colombia. Address: Cra 1 # 18A-12. Phone Number: (571) 3394949 Ext. 3900.

[2] Facultad de Medicina, Pontificia Universidad Javeriana and Hospital Universitario San Ignacio, Bogotá, Colombia. Address: Cra. 7 No. 40-62. Phone number: (571) 320 8320 Ext. 2745 – 2777.

[3] Clínica Abood-Shaio, Bogotá, Colombia. Address: Dg 115a # 70c-75. Phone number: (571) 593 8210.

[4] Grupo de Inmunobiología y Biología Celular, Facultad de Ciencias, Pontificia Universidad Javeriana, Bogotá, Colombia. Address: Carrera 7 No. 43-82 – Building Carlos Ortíz. Phone number: (571) 320 8320 Ext. 4060 – 4134.

[5] Laboratorio de Parasitología Molecular, Facultad de Ciencias, Pontificia Universidad Javeriana, Bogotá, Colombia. Address: Carrera 7 No. 43-82 - Building Carlos Ortíz. Phone number: (571) 320 8320 Ext. 4060 – 4134.

[#a] Current address: INSERM UMRS872, Cancer, Immune Control and Escape, Cordeliers Research Center, Paris, France.

*** Corresponding author:**

John Mario González MD, PhD. School of Medicine, Universidad de los Andes, Bogotá D.C. Cra 1 # 18A-12. Zip code: 111711. Phone Number: 57 (1) 3394949 ext. 3900.

E-mail: johgonza@uniandes.edu.co (JMG)

Table S1. Comparison of membrane TNF-α and CD154 expression *among ex vivo* and frozen cells.

Asymptomatic Chagasic Patients						
***T. cruzi* lysate Stimulus**						
	CD4+ T cells		P- value	CD8+ T cells		P-value
	Ex vivo	Frozen		*Ex vivo*	Frozen	
mTNF-α+ expression (Median & IQR, %)	1.90 (1.23-1.91)	1.76 (1.4-2.15)	0.99	2.16 (2.07-2.74)	2.08 (1.87-2.26)	0.56
CD154 expresssion (Median & IQR, %)	1.18 (1.04-1.40)	1.02 (0.54-1.61)	0.55	1.02 (0.65-1.07)	1.02 (0.65-1.07)	0.89

Symptomatic Chagasic Patients						
***T. cruzi* lysate Stimulus**						
	CD4+ T cells		P- value	CD8+ T cells		P-value
	Ex vivo	Frozen		*Ex vivo*	Frozen	
mTNF-α+ expression (Median & IQR, %)	1.82 (1.36-2.86)	1.55 (1.26-1.66)	0.37	4.05 (2.27-4.83)	3.59 (3.14-3.75)	0.88
CD154 expresssion (Median & IQR, %)	2.91 (1.82-3.79)	2.31 (2.06-4.13)	>0.99	1.77 (0.97-1.93)	1.26 (1.11-1.62)	0.98

Healthy Controls						
***T. cruzi* lysate Stimulus**						
	CD4+ T cells		P-value	CD8+ T cells		P-value
	Ex vivo	Frozen		*Ex vivo*	Frozen	
mTNF-α+ expression (Median & IQR, %)	0.42 (0.35-0.63)	0.41 (0.31-0.47)	0.65	0.70 (0.55-0.74)	0.32 (0.26-0.51)	0.50
CD154 expresssion (Median & IQR, %)	0.10 (0.05-0.14)	0.07 (0.06-0.10)	0.99	0.19 (0.11-0.46)	0.14 (0.11-0.24)	0.64

P-value obtained by Mann-Whitney analysis
