# Supplementary material for: T cells responding to Trypanosoma cruzi detected by membrane TNF‐α and CD154 in chagasic patients
Source: Immun Inflamm Dis. 2017 Oct 1;6(1):47–57. doi: 10.1002/iid3.197 (PMC5818450; doi:10.1002/iid3.197)
Supplement: Supplementary file 4 — Table S2. Membrane TNF‐α and CD154 expression in T cell subsets after T. cruzi lysate stimulation. [file IID3-6-47-s004.pdf]

T cells responding to *T. cruzi* detected by membrane TNF-α and CD154 in chagasic patients

Juan G. Ripoll[1], Nicolás A. Giraldo[1#a], Natalia I. Bolaños[1], Nubia Roa[2], Fernando Rosas[3], Adriana Cuéllar[4], Concepción J. Puerta[5], John M. González[1*]

[1] Grupo de Ciencias Básicas Médicas, Facultad de Medicina, Universidad de los Andes, Bogotá, Colombia. Address: Cra 1 # 18A-12. Phone Number: (571) 3394949 Ext. 3900.

[2] Facultad de Medicina, Pontificia Universidad Javeriana and Hospital Universitario San Ignacio, Bogotá, Colombia. Address: Cra. 7 No. 40-62. Phone number: (571) 320 8320 Ext. 2745 – 2777.

[3] Clínica Abood-Shaio, Bogotá, Colombia. Address: Dg 115a # 70c-75. Phone number: (571) 593 8210.

[4] Grupo de Inmunobiología y Biología Celular, Facultad de Ciencias, Pontificia Universidad Javeriana, Bogotá, Colombia. Address: Carrera 7 No. 43-82 – Building Carlos Ortíz. Phone number: (571) 320 8320 Ext. 4060 – 4134.

[5] Laboratorio de Parasitología Molecular, Facultad de Ciencias, Pontificia Universidad Javeriana, Bogotá, Colombia. Address: Carrera 7 No. 43-82 - Building Carlos Ortíz. Phone number: (571) 320 8320 Ext. 4060 – 4134.

[#a] Current address: INSERM UMRS872, Cancer, Immune Control and Escape, Cordeliers Research Center, Paris, France.

*** Corresponding author:**

John Mario González MD, PhD. School of Medicine, Universidad de los Andes, Bogotá D.C. Cra 1 # 18A-12. Zip code: 111711. Phone Number: 57 (1) 3394949 ext. 3900.

E-mail: johgonza@uniandes.edu.co (JMG)

Table S2. Membrane TNFα and CD154 expression in T cell subsets after *T. cruzi* lysate stimulation.

	Asymptomatic Chagasic patients	Symptomatic Chagasic patients	$^{\Delta}$ ***P*-value**	Healthy controls	Non-chagasic cardiomyopathy	$^{\Omega}$***P*-value**
			**Membrane TNF-α expression with *T. cruzi* lysate**			
CD4+ T cells % (Median & IQR)[#]	1.90 (1.23-2.03)	1.66 (1.21-2.13)	0.7939	0.42 (0.32-0.53)	0.74 (0.68-1.06)	<0.0001*
CD8+ T cells	2.10 (2.05-2.74)	3.67 (2.58-4.07)	0.0148*	0.55 (0.28-0.72)	0.84 (0.60-1.22)	<0.0001*
			**CD154 (CD40L) expression with *T. cruzi* lysate**			
CD4+ T cells % (Median & IQR)	1.05 (1.02-1.61)	2.87 (1.82-4.10)	0.0148*	0.07 (0.06-0.13)	0.09 (0.07-0.12)	<0.0001*
CD8+ T cells	1.07 (0.89-1.27)	1.44 (1.06-1.89)	0.1694	0.16 (0.12-0.36)	0.30 (0.21-0.46)	<0.0001*

[#] Data are shown as median and interquartile range (IQR)

$^{\Delta}$ Comparisons between asymptomatic and symptomatic chagasic patients were done using the Mann-Whitney U-test.

$^{\Omega}$ Comparisons between all groups were done using Kruskal-Wallis test.

* Statistically significant, *P*-value < 0.05.
